# MicroRNA-223 Regulates Granulopoiesis but Is Not Required for HSC Maintenance in Mice

**DOI:** 10.1371/journal.pone.0119304

**Published:** 2015-03-20

**Authors:** Maria C. Trissal, Ricardo A. DeMoya, Amy P. Schmidt, Daniel C. Link

**Affiliations:** Division of Oncology, Department of Medicine, Washington University School of Medicine, St. Louis, Missouri, United States of America; Cincinnati Children's Hospital Medical Center, UNITED STATES

## Abstract

*MIR233* is genetically or epigenetically silenced in a subset of acute myeloid leukemia (AML). *MIR223* is normally expressed throughout myeloid differentiation and highly expressed in hematopoietic stem cells (HSCs). However, the contribution of *MIR223* loss to leukemic transformation and HSC function is largely unknown. Herein, we characterize HSC function and myeloid differentiation in *Mir223* deficient mice. We show that *Mir223* loss results in a modest expansion of myeloid progenitors, but is not sufficient to induce a myeloproliferative disorder. Loss of *Mir223* had no discernible effect on HSC quiescence, long-term repopulating activity, or self-renewal capacity. These results suggest that *MIR223* loss is likely not an initiating event in AML but may cooperate with other AML associated oncogenes to induce leukemogenesis.

## Introduction

MiRNAs regulate gene expression by targeting semi-complimentary messenger RNAs for post-transcriptional silencing [1]. While somatic point mutations [2] and copy number alterations [3] involving miRNA genes are uncommon in AML, we recently reported the hemizygous loss of the *MIR223* gene in a male patient with therapy-related AML [3]. Additionally, *MIR223* has been shown to be epigenetically silenced by AML-ETO [4] and down-regulated in several blood cancers [5]. Although these studies collectively show that *MIR223* is commonly silenced in leukemia, the contribution of *MIR223* loss to disease pathogenesis is unclear. A previous study of *Mir223* deficient mice suggested that miRNA-223 negatively regulates myeloid progenitor proliferation [6]. However, miRNA-223 is highly expressed in HSCs [7,8] and its contribution to HSC function has not been rigorously assessed. As HSCs and their properties are fundamentally linked to leukemogenesis [9,10], we characterized the effect of *Mir223* deletion on HSC function to investigate how loss of this gene may contribute to normal and malignant hematopoiesis.

## Methods

### Mice

Sex- and age-matched wild-type, *Mir223* ^*-/Y*^ and female *Mir223* ^*-/-*^ mice on a C57BL/6 background were obtained form The Jackson Laboratory and maintained under standard pathogen free conditions. Mice were euthanized using carbon dioxide in a chamber using compressed gas. All mouse experiments were approved by the Washington University Animal Care and Use Committee (number 20122012).

### Blood and bone barrow analysis

Peripheral blood and bone marrow were collected as described previously [11]. Complete blood counts (CBCs) were obtained using a Hemavet 950 FS automated cell counter (Drew Scientific). Bone marrow nucleated cells were quantified using a Cellometer Auto 2000 Cell Viablity Counter (Nexcelom).

### Flow Cytometry

Bone marrow and peripheral blood was processed for flow cytometry as previously described. [12]. The following antibodies were used (all from eBiosciences unless otherwise indicated): Gr-1 (RB6–8C5), B220 (RA3–6B2), CD3e (145–2C11), Ter119 (TER-119), Sca-1 (D7), c-kit (2B8), CD34 (RAM34), FcγR (93), CD150 (Biolegend, TC15–12F12.2), CD41 (MWReg30), and Flt3 (A2F10).

Cell cycle analysis was performed as previously described [13]. In brief, bone marrow cells were stained for the indicated surface markers, fixed using the BD cytofix/cytoperm kit (BD), blocked with 5% goat serum and stained with mouse anti-human Ki-67 (clone B56; BD Pharmingen). After washing, cells were resuspended in DAPI-containing FACS buffer. **All** cells were analyzed on a Gallios flow cytometer (Beckman Coulter).

### 5-Fluorouracil Stress Response

Mice were given a single 150 mg/kg dose of 5-fluorouracil (5-FU) by intraperitoneal injection. Peripheral blood was analyzed prior to treatment and 7, 9, 11, 14, 17, and 35 days after 5-FU administration.

### Bone Marrow Transplantation

Bone marrow transplantation was performed as previously described [13]. In brief, bone marrow from Ly5.2 wild-type or *Mir223* deficient mice was mixed at a 1:1 ratio with competitor bone marrow from Ly5.1 wild-type mice. Cells were retro-orbitally injected at 4x10^6^ cells/recipient into congenic Ly5.1/Ly5.2 mice conditioned with 1,000 cGy from a ^137^Cesium source. For serial transplantation studies, bone marrow from primary recipients was harvested at least 6 weeks after transplantation, pooled, and transplanted into lethally irradiated Ly5.1 secondary recipients.

### Statistical Analysis

Unpaired student's t-test was used for cell cycle analysis and all lineage, precursor, progenitor, and stem cell population data; student's t-test with Welch's correction was used as necessary when variances were significant between groups. 5-FU, competitive repopulation, and serial transplantation studies were analyzed using 2-way ANOVA with Bonferroni post-test analysis.

## Results and Discussion

**Fig 1:**
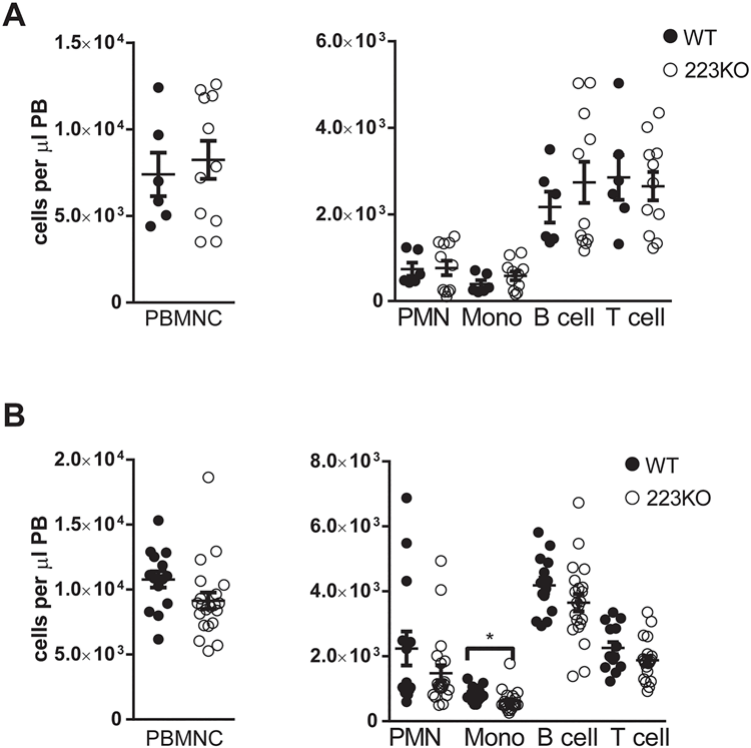
No gender specific alterations in hematopoiesis are present in *Mir223* deficient mice. (A and B) Peripheral blood from 8–12 week old wild-type and (A) female *Mir223* ^-/Y^ or (B) male *Mir223* ^-/-^ mice was analyzed at 8–12 weeks of age. The absolute number of peripheral blood mononuclear cells (PBMNC), neutrophils (PMN, Gr-1^Hi^CD115^-^), monocytes (Mono, Gr-1^INT^CD115^+^), B cells (B220^+^), and T cells (CD3e^+^) are shown. All data represent the mean ± SEM. *p<0.05.

**Fig 2:**
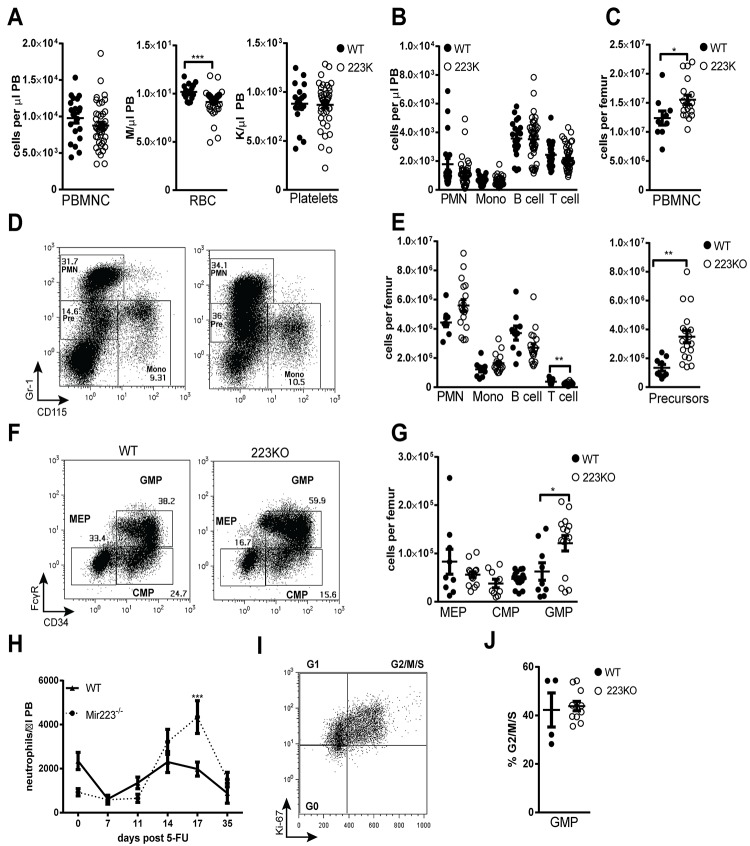
Loss of *Mir223* is associated with minimal perturbations of basal granulopoiesis. (A) Peripheral blood from male and female wild-type and *Mir223* deficient mice (223KO) was analyzed at 8–12 weeks of age. (B) Absolute number of neutrophils (PMN, Gr-1^Hi^CD115^-^), monocytes (Mono, Gr-1^INT^CD115^+^), B cells (B220^+^), and T cells (CD3e^+^) in peripheral blood. (C) Bone marrow mononuclear cell count (BMMNC). (D) Representative plots showing the gating strategy to identify mature neutrophils, granulocyte precursors (Pre, Gr-1^INT^CD115^-^) and monocytes. (E) Bone marrow lineage and granulocyte precursor data. (F) Representative plots showing the gating strategy to identify megakaryocyte-erythroid progenitors (MEP, CD34^-^FcγR^-^), common myeloid progenitors (CMP, CD34^+^FcγR^-^) or granulocyte-macrophage progenitors (GMP, CD34^+^FcγR^+^); data are gated on lineage^-^c-kit^+^sca-1^-^ cells. (G) Progenitor data.

**Fig 3:**
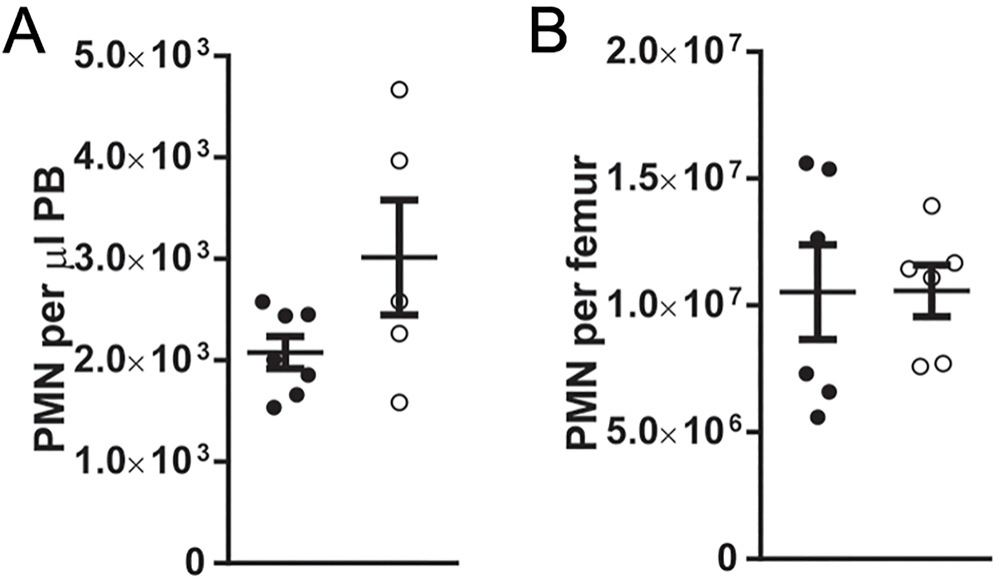
Neutrophils counts are normal in aged *Mir223* deficient mice. (A and B) Wild-type and *Mir223* deficient (223KO) mice at least 6 months old were analyzed for (A) peripheral blood or (B) bone marrow neutrophil counts. The absolute number of neutrophils (PMN, Gr-1^Hi^CD115^-^) is shown. All data represent the mean ± SEM.

We utilized an established mouse model of constitutive *Mir223* deletion to investigate the effects of miRNA-223 loss on hematopoiesis. These mice were originally characterized on a mixed strain C57BL/6 x 129Sv/Jae background and developed a myeloproliferative syndrome characterized by neutrophilia and pulmonary inflammation [6]. To eliminate the potential confounding impact of strain differences on HSC function, we analyzed mice back-crossed onto a C57BL/6 background. Peripheral blood and bone marrow counts were similar between male and female *Mir223* deficient mice (Fig. 1 and data not shown); thus, data were pooled with respect to gender. Peripheral blood counts were normal except for a modest reduction in red blood cells in *Mir223* deficient mice (Fig. 2A). Surprisingly, neutrophil counts were similar between *Mir223* deficient and control mice (Fig. 2B). Bone marrow cellularity was modestly increased in *Mir223* deficient mice (Fig. 2C). Whereas the number of mature neutrophils in the bone marrow was comparable to wild-type mice, a significant increase in Gr-1^INT^CD115^-^ granulocytic precursors was observed in *Mir223* deficient mice (Figs. 2D and 2E). A bimodal distribution of granulocyte-macrophage progenitors (GMPs) number in the bone marrow was observed in *Mir223* deficient mice (Figs. 2F and 2G). Whereas, consistent with a prior study [6], most *Mir223* deficient mice had an elevated number of GMP, in a subset it was normal. No association between gender, age, or clinical infection and GMP number was observed. Similar to young mice, no increase in neutrophils in the blood or bone marrow was observed in aged (greater than 6 month old) mice (Fig. 3). The lack of neutrophilia in our cohort contrasts with original observations of *Mir223* deficient mice on a mixed genetic background. The discrepancy is most likely due to strain differences, but it also is possible that differences in the microbiome between mouse facilities may contribute. Overall, these data suggest that loss of miRNA-223 results in a subtle increase in basal granulopoiesis in mice.

**Fig 4:**
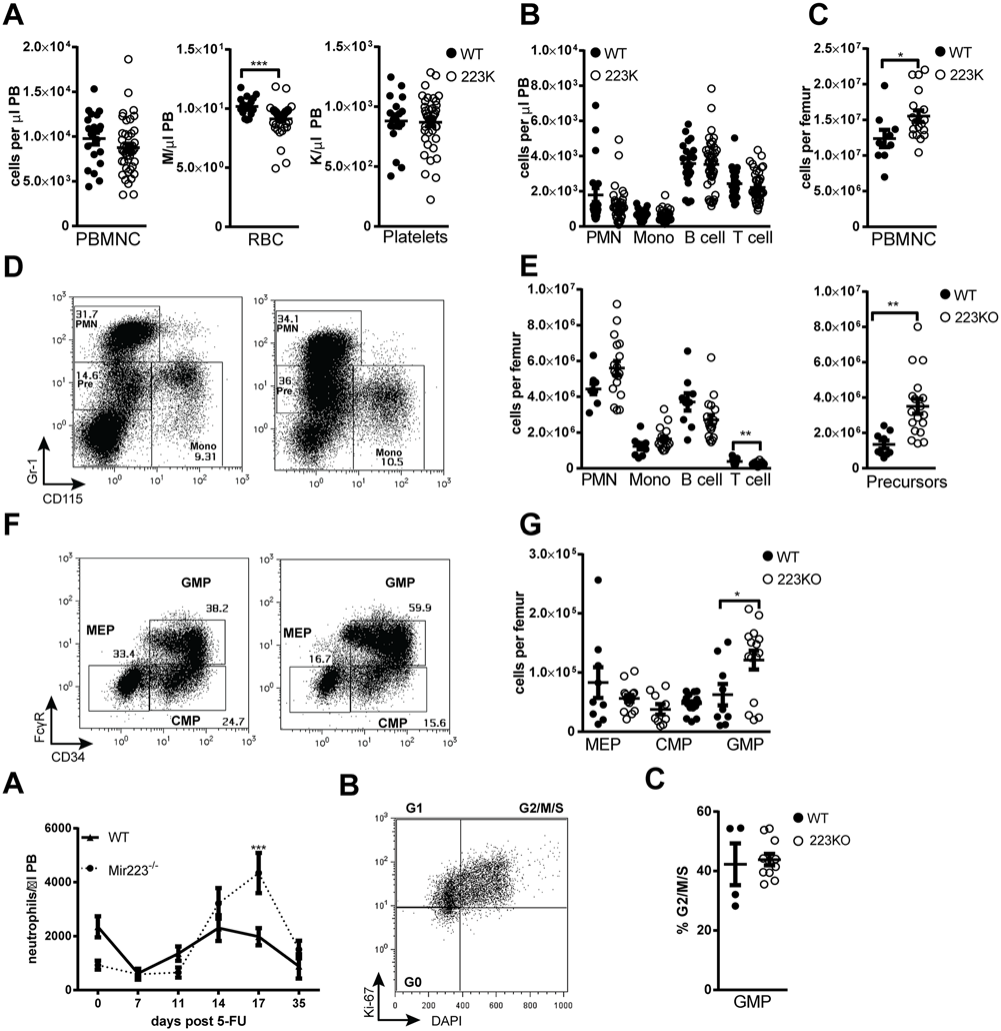
Loss of *Mir223* is associated with an increased stress granulopoiesis response. (A) Wild-type and *Mir223* deficient mice (223KO) were treated with 150 mg/kg of 5-FU and neutrophil counts were followed for 35 days (n = 8–10 mice per cohort from two independent experiments). (B) Representative plots showing Ki-67 and DAPI staining of GMPs. Harvested on day 14 following 5-FU. (C) Shown is the percentage of GMPs in the G2/M/S phase of the cell cycle. All data represent the mean ± SEM. *p<0.05, **p<0.005, ***p<0.001.

To evaluate stress granulopoiesis, neutrophil response to the myelosuppressive agent 5-FU was assessed (Fig. 4A). As expected, 5-FU induced a transient neutropenia that was followed by a rebound neutrophilia in wild-type mice. A similar degree of neutropenia was induced by 5-FU in *Mir223* deficient mice. However, *Mir223* deficient mice displayed exaggerated rebound neutrophil production. Since 5-FU primarily affects dividing cells, we assessed the cell cycle status of granulocyte progenitors. However, no significant difference in cycling cells was observed (Figs. 4B and 4C). Given the kinetics of the neutrophil recovery (peak 17 days after 5-FU), it is likely that the expanded pool of GMP is responsible for the exaggerated neutrophil response.

**Fig 5:**
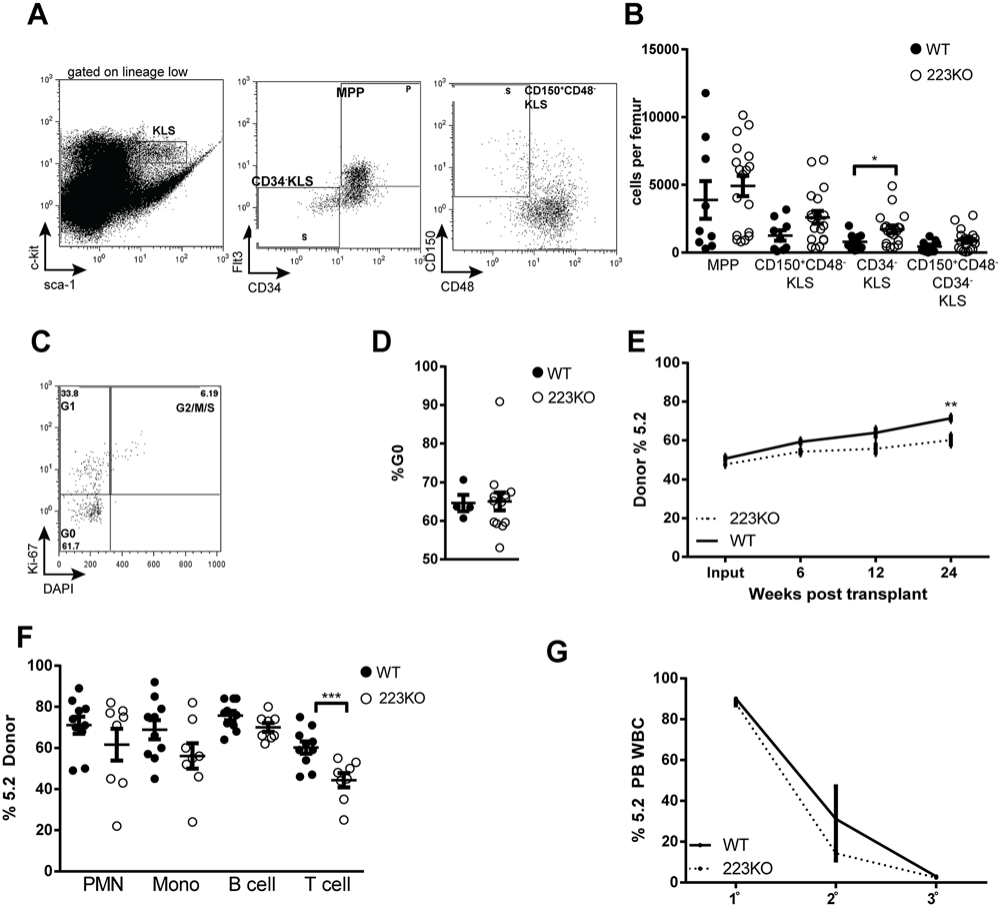
Loss of *Mir223* is not associated with alterations in HSC number or function. (A) Representative plots showing the gating strategy to identify multipotent progenitors (MPP, CD34^+^Flt3^+^), CD34^-^KLS (CD34^-^Flt3^-^KLS), or CD150^+^CD48^-^KLS cells. (B) Progenitor data for 8–12 week old wild-type (WT) or pooled male and female Mir223 deficient mice (223KO). (C) Representative plots showing cell cycle analysis of CD150^+^CD48^-^KLS cells. (D) CD150^+^CD48^-^KLS cell cycle data. (E) Wild-type or *Mir223* deficient bone marrow (Ly5.2) was mixed at a 1:1 ratio with wild-type (Ly5.1) competitor bone marrow and transplanted into irradiated recipients (Ly5.1/5.2). Shown is the percentage of Ly5.2 donor cells (n = 9–10 mice from two independent experiments). (F) Lineage distribution in the bone marrow 24 weeks after transplantation. (G) Serial transplants were performed with either Ly5.2 wild-type or *Mir223* deficient donor marrow. The percentage of donor Ly5.2 cells in the blood 6–8 weeks after transplantation is shown. (n = 4–5 mice per cohort at each time point). All data represent the mean ± SEM. *p<0.05, **p<0.005, ***p<0.001.

We next quantified hematopoietic progenitors in the bone marrow by flow cytometry; phenotypic HSCs were identified either as CD34^-^ c-kit^+^ lineage^-^ Sca^-^ (KLS) cells or as CD150^+^CD48^-^ KLS cells (Figs. 5A and 5B) [14]. Although there was a modest increase in CD150+ CD48^-^ KLS cells, no difference in multipotent-progenitors (MPP), CD34^-^ KLS cells or dormant HSCs (Flt3^-^ CD34^-^ CD150^+^ CD48^-^ KLS cells) was observed (Fig. 5B). Moreover, the percentage of CD150^+^ KSL cells in the G0 phase of the cell cycle was similar to controls (Figs. 5C and 5D), suggesting that miR-223 is not required to maintain HSC quiescence. Competitive repopulation assays showed that *Mir223* deficient HSCs had similar long-term repopulating activity as control HSCs (Fig. 5E). Likewise, analysis of the contribution of *Mir223* deficient cells to the different lineages revealed no myeloid bias, although a modest reduction to T cells was observed (Fig. 5F). Finally, serial transplantation assays showed that self-renewal capacity is normal in *Mir223* deficient HSCs (Fig. 5G). Thus, *Mir223* deficient HSCs have normal long-term repopulating activity, self-renewal capacity, and quiescence, showing that miR-223 is not required for HSC maintenance.

Collectively, these data show that miR-223 loss results in a modest expansion of myeloid progenitors without significant effects on HSCs. This contrasts with the HSC exhaustion that is observed in mice with a myeloproliferative disorder caused by expression of activating mutations of *JAK2* or *FLT3 [15,16]*. The pathways leading to myeloid expansion associated with activating mutations of tyrosine kinases genes and miR-223 loss are likely to be distinct. However, it also is possible that the severity of myeloid expansion determines whether HSCs are affected. In support of the latter possibility, mice carrying a *D835Y FLT3* mutation have a less aggressive myeloproliferative phenotype compared with *FLT3-ITD*, and HSC loss is only seen in the *FLT3-ITD* mice [17]. Our data show that deletion of *Mir223* is not sufficient to induce a myeloproliferative disorder in mice, suggesting that loss of this gene is likely not an initiating event in AML. MiR-223 silencing is often seen in association with specific mutations, such as AML-ETO. Whether miR-223 loss cooperates with AML-ETO or other oncogenes to induce AML will require further study.
